# Enterprise Data Sharing with Privacy-Preserved Based on Hyperledger Fabric Blockchain in IIOT’s Application

**DOI:** 10.3390/s22031146

**Published:** 2022-02-02

**Authors:** Chin-Ling Chen, Jiaxin Yang, Woei-Jiunn Tsaur, Wei Weng, Chih-Ming Wu, Xiaojun Wei

**Affiliations:** 1 School of Computer and Information Engineering, Xiamen University of Technology, Xiamen 361024, China; clc@mail.cyut.edu.tw (C.-L.C.); wwweng@xmut.edu.cn (W.W.); xjwei@xmut.edu.cn (X.W.); 2 School of Information Engineering, Changchun Sci-Tech University, Changchun 130600, China; 3 Department of Computer Science and Information Engineering, Chaoyang University of Technology, Taichung 41349, Taiwan; 4 Computer Center, National Taipei University, New Taipei City 237303, Taiwan; 5 School of Civil Engineering and Architecture, Xiamen University of Technology, Xiamen 361024, China; chihmingwu@xmut.edu.cn

**Keywords:** Chaincode, data security sharing, IPFS, Industrial Internet of Things (IIoT), Hyperledger Fabric blockchain, privacy-preserved

## Abstract

Internet of Things (IoT) technology is now widely used in energy, healthcare, services, transportation, and other fields. With the increase in industrial equipment (e.g., smart mobile terminals, sensors, and other embedded devices) in the Internet of Things and the advent of Industry 4.0, there has been an explosion of data generated that is characterized by a high volume but small size. How to manage and protect sensitive private data in data sharing has become an urgent issue for enterprises. Traditional data sharing and storage relies on trusted third-party platforms or distributed cloud storage, but these approaches run the risk of single-node failure, and third parties and cloud storage providers can be vulnerable to attacks that can lead to data theft. To solve these problems, this paper proposes a Hyperledger Fabric blockchain-based secure data transfer scheme for enterprises in the Industrial Internet of Things (IIOT). We store raw data in the IIoT in the InterPlanetary File System (IPFS) network after encryption and store the Keyword-index table we designed in Hyperledger Fabric blockchain, and enterprises share the data by querying the Keyword-index table. We use Fabric’s channel mechanism combined with our designed Chaincode to achieve privacy protection and efficient data transmission while using the Elliptic Curve Digital Signature Algorithm (ECDSA) to ensure data integrity. Finally, we performed security analysis and experiments on the proposed scheme, and the results show that overall the data transfer performance in the IPFS network is generally better than the traditional network, In the case of transferring 5 MB file size data, the transmission speed and latency of IPFS are 19.23 mb/s and 0.26 s, respectively, and the IPFS network is almost 4 times faster than the TCP/IP network while taking only a quarter of the time, which is more advantageous when transferring small files, such as data in the IIOT. In addition, our scheme outperforms the blockchain systems mainly used today in terms of both throughput, latency, and system overhead. The average throughput of our solution can reach 110 tps (transactions are executed per second), and the minimum throughput in experimental tests can reach 101 tps.

## 1. Introduction

### 1.1. Background

In recent years, with the rapid development of the Industrial Internet of Things (IIoT), the increase in productivity has also resulted in a significant challenge-data explosion. Enterprises in the industrial IoT use smart portable mobile terminals (e.g., drones, smartphones, electronic watches), sensors (e.g., infrared sensors, laser scanners, gyroscopes), and other large embedded devices (e.g., magnetic resonance imaging devices, traffic lights, avionics) to collect data, which is mostly unstructured and difficult to store and is maintained in traditional relational databases. Moreover, the significant amount of data also poses a challenge to IIOT terminal devices with limited computing and storage capacity. Unfortunately, the current industrial IoT still lacks a unified data management service due to the adoption of different data management systems among enterprises. In addition, the replicable and easily disseminated nature of data makes it difficult to trace the data shared among enterprises [1]. Moreover, enterprises store a large amount of data in third-party cloud storage platforms. This approach is at risk of single-node failure, and once the cloud storage server is attacked, there is a risk of data leakage, which brings serious asset loss to enterprises.

Several incidents related to the loss of stored data have already occurred in 2021 alone. Examples include the database breach of Ubiquiti, one of the world’s largest IoT technology providers [2], the database breach of Société Internationale de Télécommunications Aéronautiques (SITA) [3], the data breach of the healthcare system IT company CaptureRx [4], and the data breach of Volkswagen and Audi, a famous car brand [5].

From the above events, the future needs a decentralized storage approach to provide data storage and sharing services for the enterprise. Fortunately, the nature of blockchain technology can provide a good solution for such decentralized storage systems. The blockchain consists of individual blocks connected by a hash function, and each block contains the hash value of the previous block, a timestamp, transaction data, etc. [6]. The blockchain can be considered a distributed ledger database, which is decentralized, open and transparent, tamperproof, and traceable, and it provides a safe and reliable storage method for enterprise data. However, each client of a blockchain system must maintain a complete copy of the block data [7], and storing a large amount of data directly in the blockchain can impose a high overhead on the client. Secondly, blocks are added to the blockchain on a time-based basis [8], and data for a product in industrial systems are often contributed by multiple participants at different points in time, and uploading them directly can place a significant load on the blockchain and may make the system congested.

Therefore, this paper proposes an enterprise data sharing scheme based on the Hyperledger Fabric [9] blockchain. Sensitive raw data collected by enterprises in IIOT are encrypted with Advanced Encryption Standard (AES) [10] and stored in the InterPlanetary File System (IPFS) [11], a peer-to-peer distributed file system that provides a high-throughput content-addressable block storage system. Then, we construct the data hash address returned by IPFS into a Keyword-index table to upload to the Hyperledger Fabric blockchain and share the data between enterprises through the Keyword-index table, which can effectively reduce the load on the blockchain network. In addition, we use Chaincode deployed in the blockchain to achieve a high degree of automation in the invocation of data, and the Elliptic Curve Digital Signature Algorithm (ECDSA) [12] to sign the messages transmitted by all parties to ensure data integrity.

In summary, our contributions are as follows.

(1) We designed a data security sharing and privacy protection framework to solve the blockchain load problem and achieve enterprise privacy protection of sensitive data while improving the scalability of the system.
(2) We designed a Keyword-index table for data sharing between enterprises and designed a Chaincode to realize the automatic call of data.
(3) Our scheme realizes mutual authentication of all parties and protection of data integrity.

### 1.2. Related Works

Few studies have focused on the use of the blockchain to share data between companies or organizations. We outlined the trends in related research, focusing on discussions that combine blockchain technology, as shown in Table 1.

Teslya et al. [13] proposed a blockchain-based IIOT trust information sharing platform, and such a combination made it possible to use the mechanisms implemented in the blockchain to solve the problems identified in the platforms for IoT. Wang et al. [14] proposed a blockchain dual-chain structure, where one chain stores the original data and the other chain stores the transaction data, combined with proxy re-encryption for reliable data sharing. The scheme proposed by Zhang et al. [15] describes in detail the implementation of data sharing in eHealth systems through federated chains, where multiple hospitals form a federated chain and use bilinear mapping to ensure secure data sharing, with a very detailed evaluation of the efficiency and cost. Ra Lee et al. [16] proposed a healthcare data-sharing framework using blockchain registries and Fast Healthcare Interoperability Resources (FHIR) technology to improve operability by storing registries on the blockchain while storing the raw data in a database. Kumar et al. [17] proposed a method for health data sharing using Hyperledger Fabric by calling chain codes and listing the specific algorithmic steps. However, the above schemes are still not perfect in terms of identity authentication and data traceability, and the communication parties do not have complete trust, and there is still the risk of data leakage.

Our scheme focuses on proposing a secure data sharing and privacy protection scheme based on blockchain and smart contract technology that allows data to be shared between authorized enterprises. We ensure that the entire process from data submission to data transfer is fully recorded in the blockchain and that ECDSA is used for data integrity protection. We use data stored independently of each other to increase the scalability of the blockchain network, reduce latency and energy costs, and improve the transmission effectiveness of the network. The perfect authentication and access control mechanism can ensure that the sensitive data of enterprises will not be leaked out and effectively protect the privacy of enterprises.

The contents of the rest of the paper are as follows: Section 2 presents some related knowledge of our study. Section 3 describes our proposed architecture and the detailed workflow. In Section 4, we analyze the security of the scheme. In Section 5, we evaluate the performance of the scheme. In Section 6, we perform an experimental test of the proposed scheme. Finally, Section 7 concludes the paper.

## 2. Preliminary

### 2.1. Elliptic Curve Digital Signature Algorithm (ECDSA)

Elliptic Curve Cryptography (ECC) [18] is a public key encryption algorithm based on elliptic curve mathematics. The main advantage of ECC is that it uses a smaller key length and provides a comparable level of security compared to the Rivest–Shamir–Adleman (RSA) encryption algorithm. ECDSA is a combination of ECC and DSA (Digital Signature Algorithm). Compared with RSA, the public key length of ECDSA is shorter and the encrypted message will be smaller, so the computation and processing time will be shorter, and the memory and bandwidth requirements will be smaller. The following is the signature and verification process of ECDSA:

Signing process: Suppose Alice wants to sign a message m, the elliptic curve parameter used is D=(p,a,b,G,n,h), Alice needs to choose a random number between [1, N−1], $d_{A}$ as Alice’s private key, and generate a public key $Q_{A}=d_{A}G$. Alice will sign according to the following steps: First, Alice needs to generate a random number k between [1, N−1]; then calculate $(x_{1},y_{1})=kG$, z=h(m), $r=x_{1}\;mod\;n$, $s=(z+d_{A}r)k^{-1}\;mod\;n$. Finally, Alice sends the ECDSA signature result (*r*,*s*) to Bob.

Verification process: Bob needs to verify after receiving the signature. The verification steps are as follows: First, verify whether (*r*,*s*) is between [1, N−1]; then, calculate the following parameters: $z^{\prime }=h(m)$, $u=1z^{\prime }s^{-1}\;mod\;n$, $u=2rs^{-1}\;mod\;n$, $(x_{1}^{\prime },y_{1}^{\prime })=u_{1}G+u_{2}Q_{A}$. Finally, check whether the equation $x_{1}^{\prime }\;mod\;n=r$ is Equality: If they are equal, Bob confirms that the signature and message sent by Alice are correct.

### 2.2. Hyperledger Fabric

Hyperledger Fabric [19] is a platform for blockchain-based distributed ledger solutions that control transactions through chain codes, based on a modular architecture that provides a high degree of confidentiality, flexibility, and scalability. The transaction process is divided into the proposal phase, endorsement phase, sorting and packaging phase, and on-chain storage phase.

**Figure 1:**
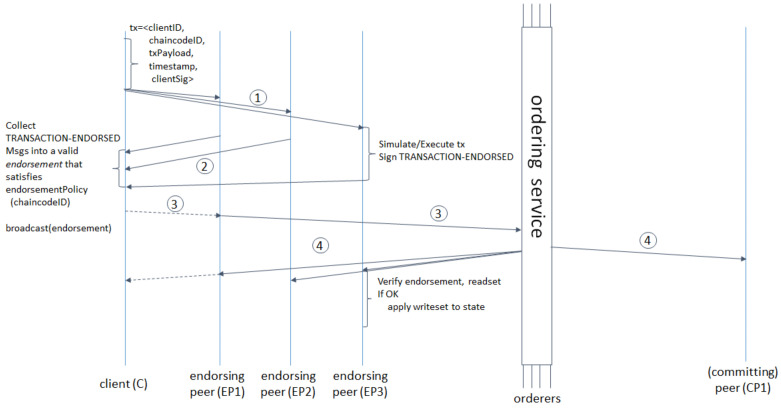
Hyperledger Fabric Transaction Flow [20].

The Hyperledger Fabric architecture is mainly composed of the following parts: Client: the blockchain network used to connect members, through the SDK to call the proposal for transactions; Certificate Authorities (CA): Certificate and public and private key issuers, mainly responsible for the identity of the member’s Management; Peers: can be divided into Leader Peer, Anchor Peer, Endorsing Peer, and Committing Peer, responsible for storing copies of the ledger and executing smart contracts (called Chaincode in Hyperledger Fabric) and approving transactions; Ordering Service (OS): responsible for collecting transaction of each channel and broadcasting to all Peers in the channel for storage on the chain. The specific workflow is shown in Figure 1:

(1) Proposal stage: The user sends the transaction to multiple Endorsing Peer through the Client.
(2) Endorsement stage: EP1, EP2, EP3 are Endorsing Peers. After receiving the proposal from the Client, it verifies and executes the endorsement, and then returns the endorsement result to the client.
(3) Sorting stage: The Client receives the endorsement results of all Endorsing Peers and compares whether they are consistent, and then sends the transaction to the Ordering Service, and the Ordering Service receives the transactions of all channels and sorts the transactions to form a block.
(4) On-chain stage: The Ordering Service broadcasts the packaged block to all Peers, and then the Peers verifies the transaction and uploads it to the blockchain.

### 2.3. Chaincode

The Chaincode in Hyperledger Fabric encapsulates the business logic used to create and modify business logic in the ledger, which can be written in different programming languages (e.g., Java, Go, and Node.js) [21]. Chaincode is created and executed by Peers to facilitate, authenticate, and enforce rules for reading, and the business logic of chain codes is defined by mutual agreement between members to read, execute, and update the current state of the ledger. When conditions are triggered, the chain code performs specific tasks, and the results of the transaction execution are submitted to the blockchain network and eventually attached to all Peers’ copies of the ledger [22].

### 2.4. InterPlanetary File System (IPFS)

The Interplanetary File System (IPFS) is a peer-to-peer distributed file system used as a distributed data storage service where the contents of the resources received by IPFS correspond to unique hashes 31. Any node in the IPFS network is independent and does not depend on other nodes, and the nodes do not need to trust each other, so there is no single point of failure as in traditional HTTP transfers. Data access will select the nearest node, greatly speeding up data transfer and reducing the storage footprint [23]. IPFS peer-to-peer transmission can effectively save network bandwidth, distributed files can effectively avoid potential DDoS attacks, and it has features, e.g., high throughput, content addressing, data anti-tampering, and de-duplication. 

### 2.5. BAN Logic

BAN Logic [24] was first proposed by Burrows et al. It is a trust-based modal logic that is usually used to prove the correctness of a protocol or scheme. During the reasoning of BAN Logic, the trust of the subjects participating in the protocol changes and evolves as the message exchange evolves. When applying BAN Logic for analysis, it is divided into the following four steps:

(1) Describe the protocol messages that are not formally described in BAN Logic notation.
(2) Identify the initial assumptions from the protocol description and describe them in BAN Logic notation.
(3) List the goals to be achieved by the protocol.
(4) Using the messages, initial conditions, and inference rules in the communication, prove whether the protocol can achieve the goal.

### 2.6. Threat Model

The threat model is an important consideration for system security issues, and the following security issues are worth analyzing in our scenario.

(1) Mutual authentication of nodes [25]: Mutual authentication refers to two parties who authenticate each other simultaneously in an authentication protocol. To ensure data security, mutual authentication is the ideal solution among authentication schemes for transmitting sensitive data. The receiver/sender must be able to confirm the legitimate identity of the sender/receiver of the message during the transmission of the message, and failure to do so will pose a great threat to data security.
(2) Data integrity [26]: Data integrity is the key to ensuring data accuracy and consistency, and to processing or retrieving data. Any accidental changes to data as a result of storage, retrieval, or processing operations can compromise data integrity. For messages transmitted in an unencrypted network environment that may be maliciously modified, data integrity may also be compromised.
(3) Data traceability [27]: Data loss due to malicious data theft by attackers, posing a serious threat to corporate assets.
(4) Non-repudiation [28]: Non-repudiation means that people cannot deny the act of sending a message and the content of the message due to the existence of some mechanism. The sender denies the message it sent, which can cause damage to the trust relationship between nodes.
(5) Resist known attack [29]: Cyber-attacks may cause data corruption or system paralysis, posing challenges to the stability and security of the system. Common attacks on blockchain networks are man-in-the-middle attacks, replay attacks, etc. For enterprises, cyber-attacks can disrupt critical infrastructure and lead to data leakage or corruption.

## 3. Proposed Scheme

### 3.1. System Architecture

**Figure 2:**
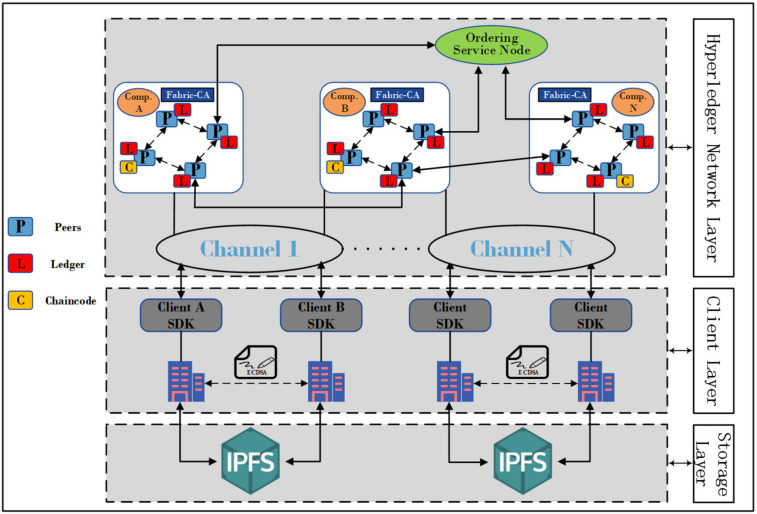
Hyperledger Fabric-based Framework for Enterprise Data Sharing and Privacy Protection.

In this article, we elaborate on the Hyperledger blockchain-based framework for enterprise data sharing and privacy protection, as shown in Figure 2. The framework is divided into three layers.

(1) Hyperledger Network Layer: This includes Peers, Ordering Service Node, Channels, and Certificate Authority (CA). The CA is responsible for issuing public and private keys and digital certificates. Administrators and Peers must be authenticated by the CA to become part of the blockchain network. The Channel is a private blockchain built based on data isolation and confidentiality. The data in the channel (e.g., Ledger information and member information) is known only to the members in the channel, and the data cannot be shared between different channels, and the channel mechanism ensures data sharing between different enterprises while protecting privacy. The Ordering Service Node only sorts and packs the transactions received in the channel and does not verify the legitimacy of the transactions, and then broadcasts the packaged transactions to all Peers in the channel. Peers are a network entity that maintains the ledger and runs the Chaincode to do read and write operations on the ledger.
(2) Client Layer: Each enterprise in the industrial IoT has an administrator who is responsible for interacting with the Hyperledger Blockchain Network. The administrator is connected to the blockchain network through the Client, which uses the SDK (Software Development Kit) to interact with the blockchain network and can access the ledger through Peers using the Chaincode, and the administrator needs to register through CA to participate in transactions in the system.
(3) Storage Layer: Enterprises that join the same channel will also join the channel’s IPFS network, which is a distributed file system for storing and sharing data, and generating a hash address for storing data, which is a key component. The administrator stores the data encrypted using AES in IPFS while constructing a Keyword-index table of the hash addresses returned by IPFS to upload to the blockchain, which greatly increases the scalability of the system. Moreover, each data transaction carries a timestamp and is permanently stored in the blockchain.

The Hyperledger Fabric blockchain can be configured with multiple Channels, and multiple enterprises can join a single Channel or join different Channels for data sharing. Enterprise administrators create their own CA in the blockchain network and then apply for a public-private key and a digital certificate using the X.509 standard from the CA to provide signatures for transactions and to endorse the results of transactions. The digital certificate contains basic information, e.g., version number, serial number, business registration number, public key, enterprise tax number, and valid time.

### 3.2. Hyperledger Fabric Detailed Transaction Information Flow

**Figure 3:**
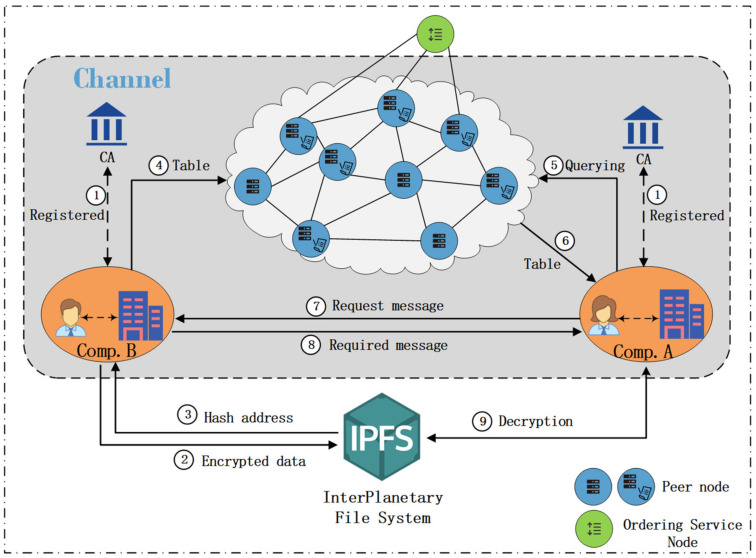
Enterprise data sharing process within Channel.

Data sharing among industrial IoT companies is realized through Channel, and different companies’ businesses may have crossover, so all parties can join the same Channel for data sharing. For example, Enterprise Administrator *A* (*A*) and Enterprise Administrator *B* (*B*) can join the same Channel for data sharing, which can be divided into four phases: registration phase, data storage phase, data query phase, and data transfer phase, and the workflow is shown in Figure 3.

Step 1. *A* and *B* need to register with the Fabric CA in Hyperledger Fabric Blockchain through the Client, and then the Fabric CA issues the public and private keys and digital certificates to the client of A and B, and the registration phase is completed.
Step 2. *B* uses the AES encryption algorithm to symmetrically encrypt and sign the sensitive and private data, and the encrypted data is saved to IPFS.
Step 3. IPFS returns the hash address of the encrypted data to the *B* Client.
Step 4. *B* Client receives the hash address and generates a Keyword-index table for the data keywords, and executes the Chaincode to add the Keyword-index table to the blockchain, and the data storage phase is completed.
Step 5. *A* sends a data access request containing keywords to the blockchain through the client.
Step 6. If the request initiated by *A* is legitimate and the queried data exists in the blockchain index directory, the blockchain network will return to *A* the required Keyword-index table containing the data hash address stored in IPFS, and the data query phase is completed.
Step 7. *A* initiates a data request to *B*, which contains *A*’s ID.
Step 8. *B* receives a request from *A*, requests *A*’s public key from the blockchain network, and verifies *A*’s request message, and then uses *A*’s public key to encrypt the AES key to form an encrypted key message and sends the message to *A*.
Step 9. After receiving the message, *A* uses its private key to decrypt it to obtain the AES key, obtains the encrypted data through the hash address provided by the Keyword-index table in IPFS, and then uses the AES key to decrypt the encrypted data to obtain the original data. The transfer phase is completed.

### 3.3. Registration Phase (Phase 1)

In this phase, enterprises joining the blockchain network for the first time need the administrator (X) to register with the CA via the client, and the registration phase proceeds as follows.

Step 1. X hashes the registration information to be submitted to obtain $h(M_{SUBMIT})$ and sends it to the CA.
Step 2. CA generates ECDSA private key $d_{X}$ based on the X and calculates $Q_{X}=d_{X}\times G$. If the identity of the registered role is verified as legitimate, the CA sends $\left(d_{X},Q_{X}\right)$ and $Cert_{X}$ to the X Client, where $Cert_{X}$ contains a unique $ID_{X}$.
Step 3. X stores $\left(d_{X},Q_{X}\right)$ and $Cert_{X}$.

### 3.4. Data Storage Phase (Phase 2)

**Figure 4:**
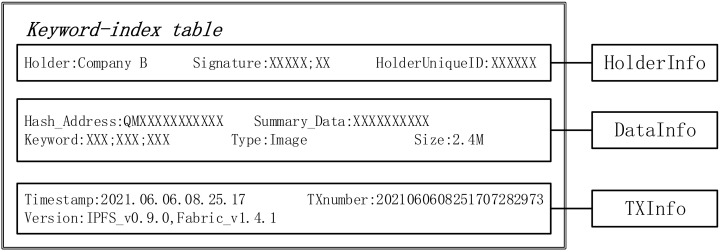
Keyword-index table structure.

In this phase, B will store the original data in IPFS after AES encryption through the client, and at the same time, construct the hash address of the encrypted data returned from IPFS to generate a *Keyword-index table* (as shown in Figure 4) for uploading to Hyperledger Fabric Blockchain (*HFB*). The workflow is shown in Algorithm 1 and can be divided into four steps.

The Keyword-index table structure is as follows:

(1) “Holder”: Name of the enterprise holding the data. “Signature”: Signature of the enterprise administrator to ensure the integrity of the data. “ID”: The unique identifier of the enterprise administrator, which is included in the certificate.
(2) “Hash_Address”: The hash address of the data, the only basis for content addressing in an IPFS network. “Summary_Data”: a brief description of the data content. “keyword”: the search basis for the data requester to query this index table in the blockchain by keyword. “size”: the size of the data. “type”: the type of the data.
(3) “Timestamp”: Indicates the time when this index table was added to the blockchain, added by Peers. “TXnumber”: transaction serial number, which is the unique value of the index table to search in the blockchain. “Version”: including IPFS version number and Fabric version number.

**Algorithm 1:** Data Storing.**Input**:
$DT_{B}$;***Step 1***:
$M_{1}$;*B* chooses a random number
$k_{B1}$;$M_{1}=(ID_{B}∥T_{1}∥DT_{B})$;**Sign**$M_{1}$; call function $Sign(M_{1},d_{B},k_{B1})$, return $\left(r_{B1},s_{B1}\right)$;$C_{B1}\leftarrow E_{SK_{B}}(M_{1})$;Send
$C_{B1},(r_{B1},s_{B1})$ to IPFS;***Step 2***: hash address;Upon receiving; check whether
$T_{NOW}-T_{1}\leq \tau ;$
**if**

$T_{NOW}-T_{1}\leq \tau$

**then**
  store
$C_{B1}$ and generating Hash_Data;  send
Hash_Data to *B*;
**end**
***Step 3***: *Keyword-index table;*Upon receiving; Generate Keyword-index table;*B* chooses a random number
$k_{B2}$;$M_{2}=(ID_{B}∥T_{2}∥Keyword-index\;table)$;**Sign**$M_{2}$; call function $Sign(M_{2},d_{B},k_{B2})$, return $(r_{B2},s_{B2});$send $M_{2}$, $\left(r_{B2},s_{B2}\right)$ to *HFB*;***Step 4***: Add to ledger;Upon receiving; check whether $T_{NOW}-T_{2}\leq \tau ;$ call function $Verify(z_{B2}{}^{\prime },r_{B2},s_{B2})$, return result;
**if**

$T_{NOW}-T_{2}\leq \tau \;$

**then**
  **if** result = “valid” **then**   call chaincode “Subfile”, add $\left(M_{2},Submit_{B}\right)$ to ledger;
**  end**

**end**

Step 1. *B* selects a random number $k_{B1}$, selects the data $DT_{B}$ to be stored, and generates the message:
  $$M_{1}=(ID_{B}∥T_{1}∥DT_{B})$$

The function $Sign(M_{1},d_{B},k_{B1})$ is called to generate the signature $\left(r_{B1},s_{B1}\right)$ for $M_{1}$ (as shown in Algorithm 2) and then uses the AES encryption algorithm to symmetrically encrypt $M_{1}$ to get $C_{B1}=E_{SK_{B}}(M_{1})$. $C_{B1}$ and $\left(r_{B1},s_{B1}\right)$ are stored in IPFS.
**Algorithm 2:** Signature and Verification of the Scheme.func *Sign* (*M* string, *d* string, *k* string)(*r* string, *s* string){(x,y)=k×G;z←h(M);$r\leftarrow x\;mod\;n,\;s\leftarrow k^{-1}(z+r\times d)\;mod\;n$;return
r,s}func *Verify* (*z* string, *r* string, *s* string, *Q* string) (result string){$u_{1}\leftarrow z\times s^{-1}\;mod\;n$$u_{2}\leftarrow r\times s^{-1}\;mod\;n$$(x^{\prime },y^{\prime })=u_{1}*G+u_{2}*Q$**if**$x^{\prime }==r\;mod\;n$**then**return “valid”**else**return “invalid”**end**}

Step 2. IPFS first checks the validity of the timestamp to prevent replay attacks, then stores the message in the IPFS network and returns the hash address to *B*.
Step 3. *B* generates a *Keyword-index table* for data keywords, and then selects a random number $k_{B2}$ to generate a message:
  $$M_{2}=(ID_{B}∥T_{2}∥Keyword-index\;table)$$

The function $Sign(M_{2},d_{B},k_{B2})$ is called to generate the signature $\left(r_{B2},s_{B2}\right)$ for $M_{2}$ and then send $M_{2}$, $\left(r_{B1},s_{B1}\right)$ to *HFB*.

**Figure 5:**
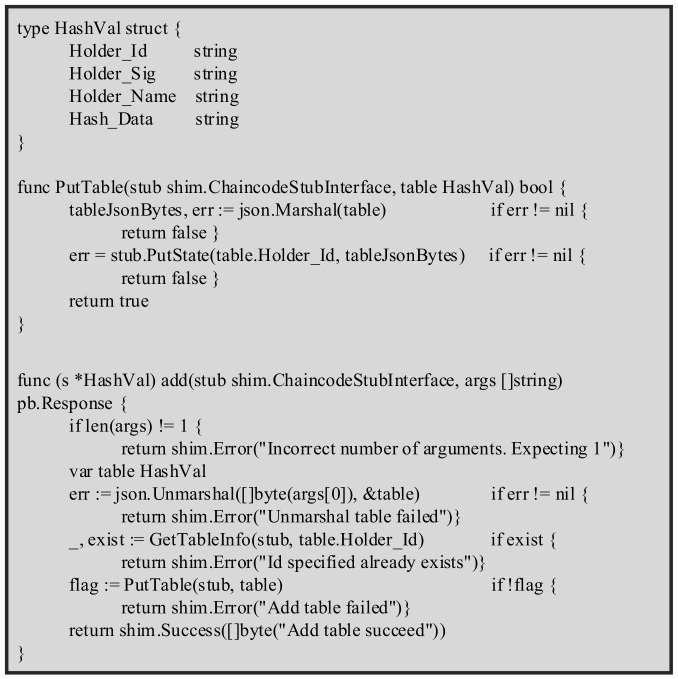
Chaincode Subfile of the proposed scheme.

Step 4. *HFB* checks the validity of the timestamp $T_{NOW}-T_{2}\leq \tau$ and then calls the function $Verify(z_{B2}{}^{\prime },r_{B2},s_{B2})$ (as shown in Algorithm 2) to verify the legitimacy of the signature. If $x_{B2}{}^{\prime }=r_{B2}\;mod\;n$, the signature is legal. Then, the Chaincode “Subfile” (as shown in Figure 5) is executed to be added $\left(M_{2},Submit_{B}\right)$ to the blockchain ledger, $Submit_{B}=h(r_{B2},s_{B2})$. The data storage phase is completed.

### 3.5. Data Query Phase (Phase 3)

Enterprises that need to query data, for example, *A*, need to submit a query request to *HFB*, and if the submitted request is legitimate, *HFB* will return the *Keyword-index table* to *A*. The workflow is shown in Algorithm 3 and can be divided into two steps.
**Algorithm 3:** Data Querying.**Input**: $M_{A-HFB}$;***Step 1***: query*A* chooses a random number
$k_{A1}$;$M_{A-HFB}=(ID_{A}∥T_{A-HFB}∥Keywords)$;call function
$Sign(M_{A-HFB},d_{A},k_{A1})$, return $\left(r_{A1},s_{A1}\right)$;send $M_{A-HFB},\;(r_{A1},s_{A1})$ to *HFB****Step 2***: returnUpon receiving; check whether
$T_{NOW}-T_{A-HFB}\leq \tau$;call function
$Verify(z_{A1}{}^{\prime },r_{A1},s_{A1})$, return result;**if**$T_{NOW}-T_{A-HFB}\leq \tau$**then**  **if** result = “valid” **then**   call chaincode “Querfile”, return *Keyword-index table* to *A*;  **end****end**

**Figure 6:**
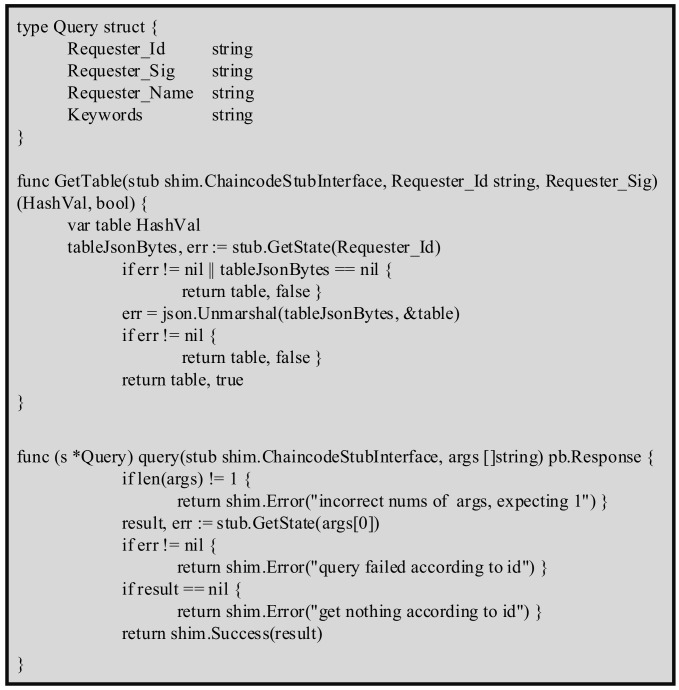
Chaincode Querfile of the proposed scheme.

Step 1. *A* selects a random number $k_{A1}$, enters keywords, and draws up a query message:
  $$M_{A-HFB}=(ID_{A}∥T_{A-HFB}∥Keyword)$$
  and calls the function $Sign(M_{A-HFB},d_{A},k_{A1})$ to generate the signature $\left(r_{A1},s_{A1}\right)$ for $M_{A-HFB}$ and then sends $M_{A-HFB}$, $\left(r_{A1},s_{A1}\right)$ to *HFB*.
Step 2. *HFB* checks the timestamp $T_{NOW}-T_{A-HFB}\leq \tau$ and calls the function $Verify(z_{A1}{}^{\prime },r_{A1},s_{A1})$ to verify the validity of the signature. If $x_{A1}{}^{\prime }=r_{A1}\;mod\;n$, the signature is legal. Then, the Chaincode “Querfile” (as shown in Figure 6) is executed to be added $\left(M_{A-HFB},Query_{A}\right)$ to the blockchain, $Query_{A}=h(r_{A1},s_{A1})$. Moreover, then, the blockchain returns the *Keyword-index table* to *A*. The data query phase is completed.

### 3.6. Data Transfer Phase (Phase 4)

*A* request $SK_{B}$ from *B*, and then the original data is obtained through $SK_{B}$. See the workflow Algorithm 4, which can be divided into 3 steps.
**Algorithm 4:** Data Transferring.**Input**: $M_{A-B}$;***Step 1***: request;*A* chooses a random number
$k_{A2}$;$M_{A-B}=(ID_{A}∥T_{A-B}∥ID_{B}∥Hash\_Data∥Txnumber);$call function
$Sign(M_{A-B},d_{A},k_{A2})$, return $\left(r_{A2},s_{A2}\right)$;$C_{A-B}\leftarrow E_{Puk_{B}}(M_{A-B})$send $C_{A-B},\;(r_{A2},s_{A2})$ to *HFB*;***Step 2***: return;Upon receiving;
$M_{A-B}=D_{Prk_{B}}(C_{A-B});$check whether $T_{NOW}-T_{A-B}\leq \tau$;call function $Verify(z_{A2}{}^{\prime },r_{A2,s_{A2})}$, return result;**if**$T_{NOW}-T_{A-B}\leq \tau$**then**  **if** result = “valid” **then**    *B* chooses a random number
$k_{B3}$;   $M_{B-A}=(ID_{B}∥T_{B-A}∥ID_{A}∥SK_{B})$   call function $Sign(M_{B-A},d_{B},k_{B3})$, return $\left(r_{B3},s_{B3}\right)$;   $C_{B2}\leftarrow E_{Puk_{A}}(M_{B-A});$   send $C_{B2},(r_{B3},s_{B3})$, to *A*;**  end****end*****Step 3***: Descrypt data;$M_{B-A}=D_{Prk_{A}}(C_{B-A})$; check whether $T_{NOW}-T_{B-A}\leq \tau$;call function $Verify(z_{B3}{}^{\prime },r_{B3},s_{B3})$, return result;**if**$T_{NOW}-T_{B-A}\leq \tau$**then**  **if** result = “valid” **then**   store $SK_{B}$; get encrypted data in IPFS;   $M_{1}=D_{SK_{B}}(C_{B1});$  **end****end**

Step 1. *A* selects a random number $k_{A2}$, and draws up the requested message:
  $$M_{A-B}=(ID_{A}∥ID_{B}∥T_{A-B}∥Hash\_Data∥TXnumber)$$
  and calls the function $Sign(M_{A-B},d_{A},k_{A2})$ to generate the signature $\left(r_{A1},s_{A1}\right)$ for $M_{A-B}$ and uses $Puk_{B}$ to encrypt the $M_{A-B}$ to obtain $C_{A-B}=E_{Puk_{B}}(M_{A-B})$. Then, send $C_{A-B}$, $\left(r_{A2},s_{A2}\right)$ to *B*.
Step 2. After receiving the requested message, *B* decrypts the message $M_{A-B}=D_{Prk_{B}}(C_{A-B})$ using $Prk_{B}$, and checks the validity of the timestamp $T_{NOW}-T_{A-B}\leq \tau$. Then, it calls the function $Verify(z_{A2}{}^{\prime },r_{A2},s_{A2})$ to verify the validity of the signature. If $x_{A2}{}^{\prime }=r_{A2}\;mod\;n$, the signature is legal. Next, *B* selects the random number $k_{B3}$ and adds $SK_{B}$ to the message:
  $$M_{B-A}=(ID_{B}∥ID_{A}∥T_{B-A}∥SK_{B})$$
  and calls the function $Sign(M_{B-A},d_{B},k_{B3})$ to generate the signature $\left(r_{B3},s_{B3}\right)$ for $M_{B-A}$. Afterward, *B* uses $Puk_{A}$ to encrypt the $M_{B-A}$ to obtain $C_{B-A}=E_{Puk_{A}}(M_{B-A})$. Then, send $C_{B-A}$, $\left(r_{B3},s_{B3}\right)$ to *A*.
Step 3. *A* decrypts the message $M_{B-A}=D_{Prk_{A}}(C_{B-A})$ using $Prk_{A}$ to obtain $SK_{B}$ and checks the validity of the timestamp $T_{NOW}-T_{B-A}\leq \tau$. Then, it calls the function $Verify(z_{B3}{}^{\prime },r_{B3},s_{B3})$ to verify the validity of the signature. If $x_{B3}{}^{\prime }=r_{B3}\;mod\;n$, the signature is legal. Afterward, *A* obtains the encrypted data $C_{B1}$ using the *Keyword-index table* in the IPFS network and decrypts the $C_{B1}$ with $SK_{B}$, $M_{1}=D_{SK_{B}}(C_{B1})$, $M_{1}=(ID_{B}∥T_{1}∥DT_{B})$. The data transfer phase is completed.

## 4. Security Analysis

### 4.1. Mutual Authentication

In this article, we use BAN Logic to demonstrate the mutual authentication of the two parties in the data transmission process, mainly to ensure that the data is not tampered with during the transfer phase. Table 2 shows syntax and semantics are associated with BAN Logic.

In the data transfer phase, the scheme mainly authenticates the legitimacy of the identity of the communicating parties, and the main objectives of the scheme are:

$$G1:\;A|\equiv A\overset{K_{A-B}}{↔}B$$

$$G2:\;A|\equiv B|\equiv A\overset{K_{A-B}}{↔}B$$

$$G3:\;B|\equiv A\overset{K_{A-B}}{↔}B$$

$$G4:\;B|\equiv A|\equiv A\overset{K_{A-B}}{↔}B$$

$$G5:\;A|\equiv ID_{B}$$

$$G6:\;A|\equiv B|\equiv ID_{B}$$

$$G7:\;B|\equiv ID_{A}$$

$$G8:\;B|\equiv A|\equiv ID_{A}$$

$$G9:\;A|\equiv SK_{B}$$

$$G10:\;A|\equiv B|\equiv SK_{B}$$

In the data transfer phase, BAN Logic is applied to generate the idealized form as follows:

$$M:A\to B\;({\left\{ID_{A},k_{A2},M_{Request}\right\}}_{Puk_{B}},<h(ID_{A},k_{A2},M_{Request}){>}_{K_{A-B}})$$

$$M:B\to A\;({\left\{ID_{B},k_{B3},SK_{B},M_{Reply}\right\}}_{Puk_{A}},<h(ID_{B},k_{B3},SK_{B},M_{Reply}){>}_{K_{A-B}})$$

The proposed scheme is analyzed and the following assumptions made:

$$A1:\;B|\equiv \#(k_{A2})$$

$$A2:\;A|\equiv \#(k_{B3})$$

$$A3:\;A|\equiv B|\Rightarrow A\overset{K_{A-B}}{↔}B$$

$$A4:\;B|\equiv A|\Rightarrow A\overset{K_{A-B}}{↔}B$$

$$A5:\;A|\equiv B|\Rightarrow ID_{B}$$

$$A6:\;B|\equiv A|\Rightarrow ID_{A}$$

$$A7:\;A|\equiv B|\Rightarrow SK_{B}$$

$$A8:\;A|\equiv \overset{Puk_{B}}{\to }B$$

$$A9:\;B|\equiv \overset{Puk_{A}}{\to }A$$

According to the assumptions and rules of BAN Logic, the main proofs of the data transfer phase are as follows:

(1) The administrator of Enterprise *B* (*B*) authenticates the administrator of Enterprise *A* (*A*).

Through *M1* and the seeing rule, we derive:

(1) $$B⊲({\left\{ID_{A},k_{A2},M_{Request}\right\}}_{Puk_{B}},<h(ID_{A},k_{A2},M_{Request}){>}_{K_{A-B}})$$

Through *M1* and the seeing rule, we derive:

(2) $$B|\equiv \#({\left\{ID_{A},k_{A2},M_{Request}\right\}}_{Puk_{B}},<h(ID_{A},k_{A2},M_{Request}){>}_{K_{A-B}})$$

Through Formula (1), A9, and the message meaning rule, we derive:

(3) $$B|\equiv A|∼({\left\{ID_{A},k_{A2},M_{Request}\right\}}_{Puk_{B}},<h(ID_{A},k_{A2},M_{Request}){>}_{K_{A-B}})$$

Through Formulas (2)–(3), and the nonce verification rule, we derive:

(4) $$B|\equiv A|\equiv ({\left\{ID_{A},k_{A2},M_{Request}\right\}}_{Puk_{B}},<h(ID_{A},k_{A2},M_{Request}){>}_{K_{A-B}})$$

Through Formula (4) and the belief rule, we derive (G4)–(G8):

(5) $$B|\equiv A|\equiv A\overset{K_{A-B}}{↔}B$$

(6) $$B|\equiv A|\equiv ID_{A}$$

Through Formula (5), A4, and the jurisdiction rule, we derive (G3):

(7) $$B|\equiv A\overset{K_{A-B}}{↔}B$$

Through Formula (5), A6, and the jurisdiction rule, we derive (G7):

(8) $$B|\equiv ID_{A}$$

(2) The administrator of Enterprise *A* (*A*) authenticates the administrator of Enterprise *B* (*B*).

Through *M2* and the seeing rule, we derive:

(9) $$A⊲({\left\{ID_{B},k_{B3},SK_{B},M_{Reply}\right\}}_{Puk_{A}},<h(ID_{B},k_{B3},SK_{B},M_{Reply}){>}_{K_{A-B}})$$

Through A2 and the freshness rule, we derive

(10) $$A|\equiv \#({\left\{ID_{B},k_{B3},SK_{B},M_{Reply}\right\}}_{Puk_{A}},<h(ID_{B},k_{B3},SK_{B},M_{Reply}){>}_{K_{A-B}})$$

Through Formula (9), A8, and the message meaning rule, we derive:

(11) $$A|\equiv B|∼({\left\{ID_{B},k_{B3},SK_{B},M_{Reply}\right\}}_{Puk_{A}},<h(ID_{B},k_{B3},SK_{B},M_{Reply}){>}_{K_{A-B}})$$

Through Formulas (10) and (11), and the nonce verification rule, we derive:

(12) $$A|\equiv B|\equiv ({\left\{ID_{B},k_{B3},SK_{B},M_{Reply}\right\}}_{Puk_{A}},<h(ID_{B},k_{B3},SK_{B},M_{Reply}){>}_{K_{A-B}})$$

Through Formula (12) and the belief rule, we derive (G2), (G6), and (G10):

(13) $$A|\equiv B|\equiv A\overset{K_{A-B}}{↔}B$$

(14) $$A|\equiv B|\equiv ID_{B}$$

(15) $$A|\equiv B|\equiv SK_{B}$$

Through Formula (13), A3, and the jurisdiction rule, we derive (G1):

(16) $$A|\equiv A\overset{K_{A-B}}{↔}B$$

Through Formula (14), A5, and the jurisdiction rule, we derive (G5):

(17) $$A|\equiv ID_{B}$$

Through Formula (15), A7, and the jurisdiction rule, we derive (G9):

(18) $$A|\equiv SK_{B}$$

Through Formulas (6), (8), (16), and (17), it can be proven that, in the proposed scheme, *A* and *B* authenticate each other. Moreover, it can also be proven that the proposed scheme can authenticate the private key of *A* and *B*.

In the proposed scheme, B authenticates A by verifying:

(19) $$x_{A2}{}^{\prime }=r_{A2}\;mod\;n$$

If it passes the verification, B authenticates the legality of A. A authenticates the B by verifying:

(20) $$x_{B3}{}^{\prime }=r_{B3}\;mod\;n$$

If it passes the verification, *A* authenticates the legality of *B*. The data transfer phase of the proposed scheme, thus, guarantees mutual authentication between *A* and *B*.

### 4.2. Data Integrity

In our scheme, the parties’ transaction data will be permanently stored in the blockchain network while we use ECDSA and AES to sign and encrypt the transactions to ensure data integrity. For example, in the data storage phase, *B* will sign and add timestamps to the Keyword-index table, and then upload it to the blockchain network, which will verify the timestamp $T_{NOW}-T_{2}\leq \tau$ and signature $\left(r_{B2},s_{B2}\right)$ upon receipt. If the data is tampered with, then $x_{B2}{}^{\prime }\neq r_{B2}\;mod\;n$, $M_{2}$ does not match $\left(r_{B2},s_{B2}\right)$, and the attacker’s attack failed. During the data transfer phase, both communicating parties also verify the signature upon receipt of the message to ensure the integrity of the data. The data uploaded to the blockchain is stored in the blocks in a chained data structure, and each block is linked to the previous block through a hash function. If an attacker wants to tamper with the data, he needs to modify the hash value of the whole chain, which is unrealistic in a decentralized network system.

### 4.3. Traceability

Every transaction data stored in the blockchain is signed and stored forever, and the data is transparent and can be publicly verified. For example, the message is uploaded to the blockchain with the signed hash $Submit_{B}$ of *B* in the data storage phase. In the data query phase, the signature hash $Query_{A}$ of *A* is uploaded to the blockchain $M_{Query}$. All members can trace the transaction process and determine whether the data in the blockchain is legitimate by verifying $Submit_{B}\overset{?}{=}h(r_{B2},s_{B2})$ and $Query_{A}\overset{?}{=}h(r_{A1},s_{A1})$.

### 4.4. Non-Repudiation

In the proposed scheme, ECDSA’s private key signature is used to achieve non-repudiation. The messages sent by all members of the system use their private keys to sign the messages. The receiver will verify the signature after receiving the message. If the verification is successful, the sender cannot deny the content of the message sent. Table 3 shows the non-repudiation of each role in the proposed scheme.

### 4.5. Resist Known Attacks

In this phase, we analyzed possible attacks against the system, including man-in-the-middle attacks and replay attacks.

### 4.6. Man-in-the-Middle Attack

The attacker tries to intercept and tamper with the message content. In our scheme, both communicating parties do not have to send their public keys to each other, and both parties can query each other’s public keys in the blockchain network, which can effectively prevent the attacker from intercepting the message and replacing the public key. For example, *A* uses *B*’s public key to encrypt the message $C_{A-B}=E_{Puk_{B}}(M_{Request})$. *B* uses *A*’s public key to encrypt the message $C_{B-A}=E_{Puk_{A}}(M_{Reply})$. The attacker does not know the private keys of the communicating parties, so he cannot decrypt the message.

### 4.7. Replay Attacks

The messages of the two communicating parties may be intercepted by the attacker, who pretends to be a legitimate sender and sends the same message to the recipient. In our scheme, a timestamp mechanism is added between two parties of arbitrary communication to prevent such attacks. For example, during the data transfer phase, *B* sends a timestamped message $M_{B-A}$ to *A*, who checks that the timestamped message $T_{NOW}-T_{B-A}\leq \tau$ is valid. Even if the attacker tampers with the timestamp data, because *B* has added a timestamp TB−A ($s_{B3}=k^{-1}(z_{B3}+r_{B3}d_{B})\;mod\;n$, $z_{B3}=h(M_{B-A})$) to the signature $\left(r_{B3},s_{B3}\right)$, A checks that the timestamp does not match the signature and the replay attack fails.

## 5. Performance Evaluation

### 5.1. Communication Cost

Table 4 shows the communication cost analysis of the proposed scheme. In the Gigabit Ethernet environment, the maximum transmission speed is 1 Gbps, and in the 10 Gigabit Ethernet environment, the maximum transmission speed is 10 Gbps. We assume that the ECDSA signature and key are 160 bits, the asymmetric encryption message is 1024 bits, the hash function operation requires 160 bits, and the length of other messages (such as ID and timestamp, etc.) is 80 bits. Taking the data transmission phase with the highest communication cost as an example, *A* needs to send two signatures, one hash, one asymmetric encrypted message, and one other message to *B*. The total size is 2 × 160 bits + 160 bits + 1024 bits + 80 bits = 1584 bits. *B* needs to send two signatures, one hash, one asymmetric encrypted message, and one other message to *A*. The total size is 2 × 160 bits + 160 bits + 1024 bits + 80 bits = 1584 bits. The total communication cost for the data transfer phase is 1584 bits + 1584 bits = 3168 bits, which takes 3.168 μs in a Gigabit Ethernet communication environment and 0.3168 μs in a 10 Gigabit Ethernet environment. These communication costs are very low, so the proposed scheme has good communication performance.

### 5.2. Computation Cost

In Table 5, we analyze the computational cost of each phase of the scheme, and we use asymmetric encryption and decryption, hashing operations, and addition, subtraction, multiplication, and division operations as the basis for the computational cost analysis. Taking the data transfer phase (phase 4) with the highest computational cost as an example, *A* requires three encryption/decryption operations, two comparison operations, five modular operations, two hash operations, eight multiplication operations, and one signature operation. *B* requires two encryption/decryption operations, two comparison operations, five modular operations, two hash operations, eight multiplication operations, and one signature operation. Thus, in our scheme, the calculation cost is acceptable.

### 5.3. Blockchain Architecture Comparison

There are currently at least four types of blockchain networks: public blockchains, private blockchains, consortium blockchains, and hybrid blockchains [30]. Private blockchains are too centralized and not suitable for data sharing between enterprises but only for resource management within a specific individual or company. We summarize the comparison between two blockchain platforms, Hyperledger Fabric, a typical representative of consortium blockchains, and Ethereum, a typical representative of public blockchains, as shown in Table 6.

From the above table, we can see that although Ethereum has advantages in fault tolerance and the transaction success rate, Hyperledger Fabric outperforms Ethereum in terms of the average transaction latency, throughput, privacy, and scalability, and the modularity and channel design of Hyperledger Fabric is more suitable for data sharing among enterprises [31].

### 5.4. Function Comparison

Table 7 shows the comparison of the previous scheme with our proposed scheme. It can be seen from the table that this scheme overcomes the shortcomings of the previous scheme.

We compare with previous studies, which, as mentioned before, have some flaws, we improve on the flaws based on the previous work. Teslya et al. [13] proposed a blockchain-based IIOT trust information sharing platform. Tis paper describes a possible way of integrating IoT and blockchain technology to solve these problems. To this end, an architecture combining the Smart-M3 information sharing platform and the blockchain platform was developed. However, it only proposes an architecture without detailed deployment and experiments. Furthermore, this paper does not discuss the security of the architecture and lacks a theoretical basis. This paper has detailed instructions on system security and experimental testing. Wang et al. [14] proposed a new data-sharing scheme based on blockchain technology, which combines the blockchain with a double-chain structure and proxy re-encryption to achieve safe and reliable data sharing. This scheme only discusses the security and complexity of the system and does not have actual experimental tests. In addition, this scheme cannot detect the source of data leakage, and the segmentation of data blocks lacks theoretical support. We experimentally test the proposed scheme, and we employ signature technology to ensure data traceability. Zhang et al. [15] proposed a blockchain-based security and privacy-preserving PHI sharing (BSPP) scheme for improving diagnosis in e-health systems. However, the scheme uploads all PHI data to the blockchain network, which undoubtedly increases the overhead of the blockchain client, and the scheme does not provide discussion on the authentication between the nodes of the Consortium chain. Our solution uses off-chain storage of data to reduce the overhead of the blockchain network, and we use ban logic proof to prove the identity security among the nodes. Ra Lee et al. [16] proposed a standards-based sharing framework SHAREChain that combines two properties to deal with reliability and interoperability issues and Kumar et al. [17] proposed a healthcare application based on a blockchain network with a Hyperledger fabric structure, but these two schemes do not discuss the security and efficiency of the system. We illustrate the safety of the proposed scheme, and the experimental results show the good efficiency of our scheme.

We propose a complete system framework focusing on the security issues of enterprise data transmission among blockchain networks. Therefore, we focus on the security issues of the system in the analysis phase. Compared to previous studies, our solution has advantages in data privacy, data protection, and data traceability, which are lacking in previous solutions, while we adopt off-chain storage of data to increase the scalability of the blockchain network and use digital signature technology to ensure the authenticity of data. Finally, the experimental results show that our scheme has good efficiency and practical prospects.

## 6. Deployment and Testing

In this section, we experimentally evaluate the proposed scheme. The HyperLedger Fabric uses Docker container technology to run the Chaincode containing the system application logic. The Fabric framework includes a certificate authority (CA), order nodes, and peer nodes. Each peer node maintains a full copy of the blockchain data, and in our scenario, the Enterprise Administrator is the peer node. Each peer node uses CouchDB to maintain the state of its ledger. All nodes are run in their own Docker containers. We deployed 6 peer nodes, 1 order node and 2 CA on a server with Intel Core i7-8700 @3.2GHz CPU and 8 GB RAM. The operating system of the physical machine is Ubuntu 18.04.2 LTS. The version of Fabric we used is v1.4.

### 6.1. Performance of File Transmission in Traditional and IPFS Network

**Figure 7:**
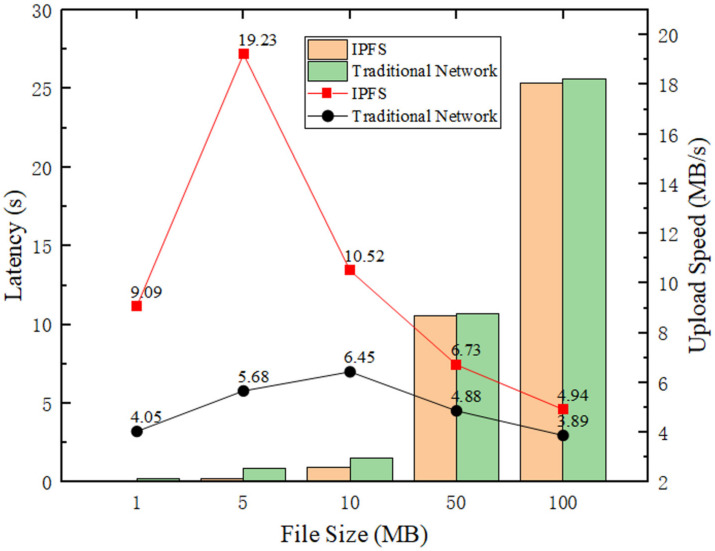
Performance comparison of file transfers in traditional and IPFS networks using different file sizes.

In this experiment, we compared the file upload performance of different file sizes in traditional TCP/IP networks and IPFS networks. Because the number of IoT devices is huge in industrial IoT networks and each device can only generate a small amount of data, we chose files of sizes 1, 5, 10, 50, and 100 MB, respectively. As can be seen from Figure 7, The latency of the IPFS network was 0.11, 0.26, 0.95, 10.55, and 25.34 s, while the latency of the TCP/IP network was 0.25, 0.88, 1.55, 10.71, and 25.65 s, respectively. In terms of transmission speed, the transmission speed of IPFS is 9.09, 19.23, 10.52, 6.73, and 4.94 MB/s while the transmission speed of TCP/IP is 4.05, 5.68, 6.45, 4.88, and 3.89 MB/s, respectively.

From the experimental results, almost all the transfer rates in the IPFS network are faster than in the TCP/IP network, and the IPFS networks are almost 4 times larger than TCP/IP networks when transferring data of 5 MB file size. Moreover, IPFS networks take less time than TCP/IP networks, which is more evident when transferring small files (File Size ≤ 10 MB), IPFS networks take one-half the time of TCP/IP networks when transferring 1 MB files, and one-quarter the time of TCP/IP networks when transferring 5 MB files. The data transfer performance in IPFS networks is generally better than that in traditional networks.

### 6.2. Throughput and Latency of Smart Contract Calling

**Figure 8:**
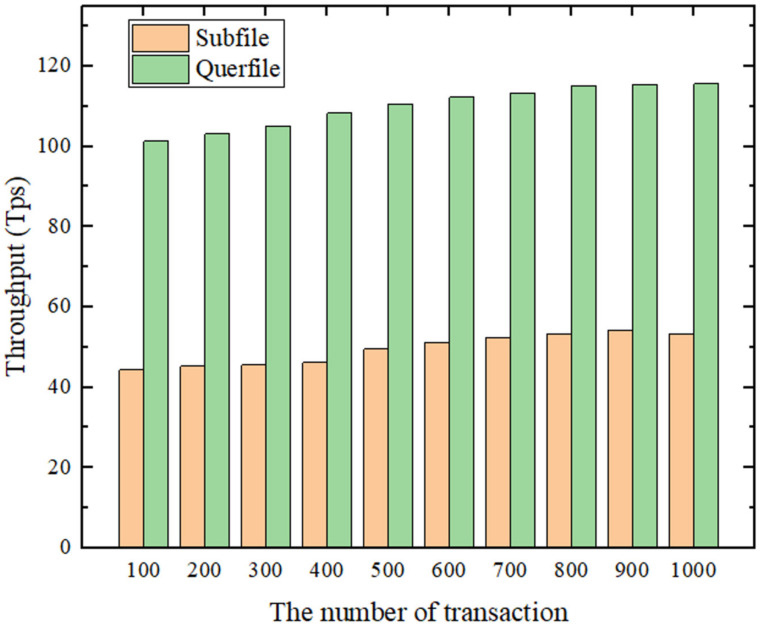
System throughput at different transaction volumes.

**Figure 9:**
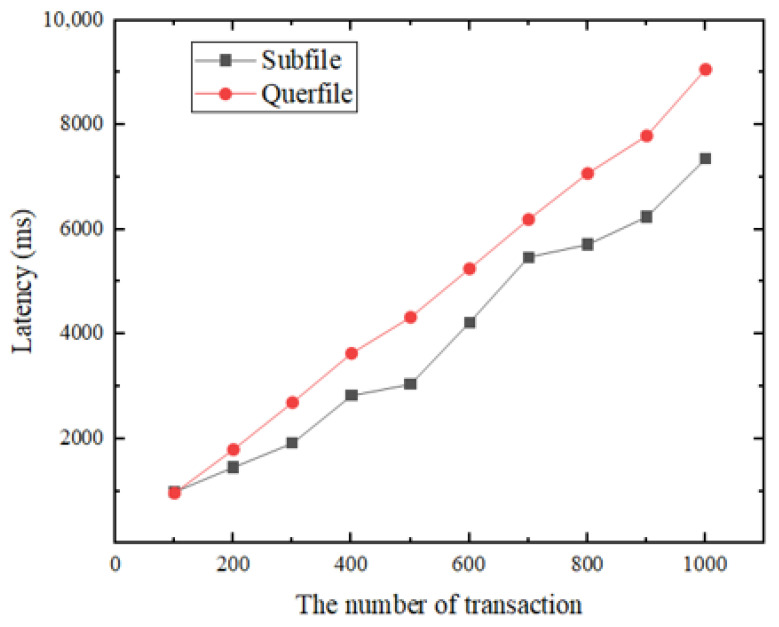
System latency at different transaction volumes.

We designed two smart contracts for the blockchain network and used throughput and transaction latency as the main performance metrics in our benchmarking. Throughput is the rate at which transactions are committed to the ledger, measured in terms of how many transactions are executed per second (tps). Latency is the time it takes from the time the application sends a transaction proposal to the time the transaction is committed to the ledger. As can be seen from Figure 8, when the block size and send rate is fixed, the TPS remains essentially constant as the number of transactions increases. “Querfile” fluctuates around 110 tps, with a minimum of 101.3 tps and a maximum of 115.6 tps; and “Subfile” fluctuates around 50 tps, with a minimum of 44. 3 tps and a maximum of 53.2 tps. In addition, as shown in Figure 9, the latency increases with the increase in the number of transactions.

### 6.3. Performance Comparison of Different Systems

**Figure 10:**
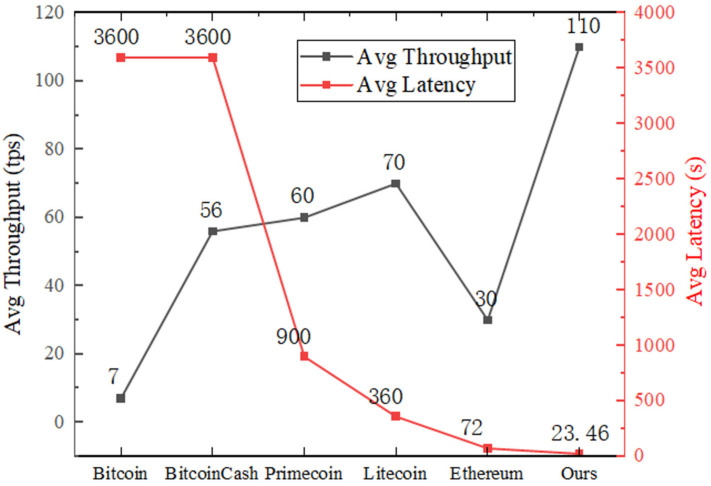
Comparison with current major blockchain systems.

To demonstrate the good performance of our proposed scheme, we compare it with other blockchain systems mainly used today: Bitcoin, Ethereum, Litecoin, BitcoinCash, and Primecoin in terms of the system transaction average latency and average throughput [32]. The sending rate, block size, and some transactions are set to 200 tps, 2 MB, and 400. Figure 10 gives the comparison results.

From the comparison, it is clear that our scheme has better performance than existing blockchain systems in terms of the average transaction latency and average throughput. In terms of throughput, the block size limits the throughput of Bitcoin to only seven transactions per second. In total, 70, 60, and 56 transactions per second are achieved for Primecoin, Litecoin, and Bitcoin Cash, respectively, while Ethereum processes about 30 transactions per second. The average throughput of our solution can reach 110 tps, and the minimum throughput in experimental tests can reach 101 tps. In terms of system overhead, since the blockchain platform used in this system is Hyperledger Fabric, it does not need to consume a lot of computational resources for mining; therefore, the overhead of our solution is extremely low.

## 7. Conclusions

To solve the data sharing and privacy protection problems brought by the rapid growth of data in industrial IoT, we proposed an enterprise privacy protection and data sharing scheme based on the Hyperledger Fabric blockchain. We focused on the security and privacy of data transmitted by all parties in industrial systems. We utilized the Hyperldeger Fabric channel mechanism to enable enterprises to share data while keeping sensitive data private, isolating data between different channels, and all transaction data will carry time stamps and be permanently stored in the blockchain ledger, and be open, transparent, and traceable. Moreover, we achieved a high degree of automation in data recall through the designed Chaincode. The under-chain storage approach can effectively increase the scalability of the system. In addition, our scheme achieves mutual authentication of all parties in the system and data integrity protection. Finally, the analysis results show that our scheme has good traceability, non-repudiation, and resistance against known cyber attacks, and good performances.

In the future, a potential research direction is how to optimize the consensus algorithm of Hyperldeger Fabric, in which the backing nodes are responsible for endorsing the legitimacy of all transaction contents and carry a large amount of sensitive transaction data. How to protect the backing nodes from attacks and enhance the processing power of backing nodes to improve the transaction speed of the whole blockchain network is one of the valuable research directions.

## Figures and Tables

**Table 1:** Comparison between the proposed and existing enterprise data sharing solutions.

Authors	Year	Objective	Technologies	Merits	Demerits
Teslya et al. [13]	2017	Proposed a blockchain-based IIOT trust information sharing platform	Blockchain, Smart Contracts, Smart-M3	It can single out the search for information in the detachment.	Leading to a slower rate of information entering the smart space
Wang et al. [14]	2018	To use blockchain double-link structure combined with proxy re-encryption for data sharing	Blockchain, Proxy re-encryption	The two chains store original data and transaction data separately, combined with proxy re-encryption to achieve reliable data sharing	There is no detailed experimental process to prove the actual effect of the program, and the safety analysis is not detailed enough
Zhang et al. [15]	2018	To realize data sharing in the electronic medical system through alliance chain	Blockchain, Bilinear maps and complexity assumptions	A detailed description of the sharing of medical data through the alliance chain, and detailed experimental analysis	Security analysis is not complete enough
Ra Lee et al. [16]	2019	To use blockchain registry and FHIR to share healthcare data	Blockchain, FHIR	Improve query efficiency by storing the registry in the blockchain and storing the original data in the database	There is no analysis process, and the plan is not complete enough
Kumar S et al. [17]	2020	To provide controlled access and secure transmission of patient health information between various healthcare organizations	Hyperledger Fabric	Investigated related literature and provided detailed algorithms and steps	No comparison with other programs, no experiments to demonstrate the actual effect of the program

**Table 2:** BAN Logic.

Symbol	Description
P|≡X	*P* trusts *X* or *P* is qualified to trust *X*
P⊲X	*P* received a message containing *X*
P|∼X	*P* has sent a message containing *X*
P|⇒X	*P* has jurisdiction over *X*
#(X)	*X* is the latest
$P\overset{K}{↔}Q$	The shared key *K* is used for communication by *P* and *Q*.
$\overset{K}{\to }P$	*P* has *X* as a public key
${\left\{X\right\}}_{K}$	The message *X* is encrypted by *K*
$<X{>}_{Y}$	This indicates that *X* combined with *Y*

**Table 3:** The non-repudiation description.

	Item	Signature Value	Sender	Receiver	Signature Verification
Phase					
**Phase 2**		$\left(r_{B2},s_{B2}\right)$	*B*	*HFB*	$Verify(z_{B2}{}^{\prime },r_{B2},s_{B2})$
**Phase 3**		$\left(r_{A1},s_{A1}\right)$	*A*	*HFB*	$Verify(z_{A1}{}^{\prime },r_{A1},s_{A1})$
**Phase 4**		$\left(r_{A2},s_{A2}\right)$	*A*	*B*	$Verify(z_{A2}{}^{\prime },r_{A2},s_{A2})$
		$\left(r_{B3},s_{B3}\right)$	*B*	*A*	$Verify(z_{B3}{}^{\prime },r_{B3},s_{B3})$

**Table 4:** Analysis of the communication cost.

	Item	Message Length	Rounds	Gigabit Ethernet (1 Gbps)	10 Gigabit Ethernet (10 Gbps)
Phase					
**Phase 1**		560 bits	2	0.56μs	0.056μs
**Phase 2**		560 bits	1	0.56μs	0.056μs
**Phase 3**		560 bits	2	0.56μs	0.056μs
**Phase 4**		3168 bits	2	3.168μs	0.3168μs

**Table 5:** Analysis of the communication cost.

	Party	*A*	*B*	*HFB*
Phase				
**Phase 2**		N/A	$1T_{E/D}$ $+4T_{Mod}$ $+2T_{H}$ $+8T_{Mul}$ $+1T_{Sym}$ $+2T_{Sig}$	$2T_{Cmp}$ $+3T_{Mod}$ $+4T_{Mul}$ $+1T_{H}$
**Phase 3**		$1T_{Cmp}$ $+2T_{Mod}$ $+1T_{H}$ $+4T_{Mul}$ $+1T_{Sig}$	N/A	$2T_{Cmp}$ $+3T_{Mod}$ $+4T_{Mul}$ $+1T_{H}$
**Phase 4**		$3T_{E/D}$ $+2T_{Cmp}$ $+5T_{Mod}$ $+2T_{H}$ $+8T_{Mul}$ $+1T_{Sig}$	$2T_{E/D}$ $+2T_{Cmp}$ $+5T_{Mod}$ $+8T_{Mul}$ $+2T_{H}$ $+1T_{Sig}$	N/A

Notes: $T_{E/D}$: Encryption/Decryption operation, $T_{H}$: Hash function operation, $T_{Mul}$: Multiplication operation, $T_{Cmp}$: Comparison of operation, $T_{Mod}$: Modular operation, $T_{Sym}$: Symmetric encryption operation, $T_{Sig}$: Signature operation.

**Table 6:** Comparison between Ethereum and Hyperledger Fabric.

	Hyperledger Fabric	Ethereum
Category	Consortium Blockchain	Public Blockchain
Description	Generic blockchain platform	Modular blockchain platform
Consensus algorithms	Practical Byzantine Fault Tolerance (PBFT)	Proof of Work (PoW)
Throughput	≥1000 TPS	≥25 TPS
Decentralization	Partial de-centralization	Completely decentralization
Fault tolerance rate	33%	50%
Success rate	Lower	Higher
Privacy	Yes	No
Authentication	Yes	No
Scalability	Yes	No
Pluggability	Yes	No

**Table 7:** Functionality comparison of previous schemes and the proposed scheme.

Authors	Year	Objective	1	2	3	4	5	6
**Teslya et al.** [13]	2017	Proposed a blockchain-based IIOT trust information sharing platform	Y	N	Y	N	N	N
**Wang et al.** [14]	2018	To use blockchain double-link structure combined with proxy re-encryption for data sharing	Y	N	N	N	Y	N
**Zhang et al.** [15]	2018	To realize data sharing in the electronic medical system through alliance chain	Y	N	N	N	Y	Y
**Ra Lee et al.** [16]	2019	To use blockchain registry and FHIR to share healthcare data	Y	Y	N	N	Y	Y
**Kumar et al.** [17]	2020	To provide controlled access and secure transmission of patient health information	Y	N	Y	N	Y	N
**Ours**	2021	Propose a solution for corporate privacy-preserved and data sharing based on Fabric blockchain	Y	Y	Y	Y	Y	Y

Notes: 1: Blockchain architecture, 2: Data integrity, 3: Mutual Authentication, 4: No-repudiation, 5: Scalability, 6: Off-chain storage; (Y) Yes; (N) No.

## Data Availability

The data used to support the findings of this study are available from the corresponding author upon request.

## Notations

$Cert_{X}$	Certificate of X issued by CA
$ID_{X}$	X’s identity
$\left(r_{X},s_{X}\right)$	Elliptic curve signature value of X
$T_{X}$	Timestamp message of X
$C_{X}$	Ciphertext sent by X
$DT_{X}$	Data sent by X
$SK_{X}$	The AES key of party X
$E_{SK_{X}}(M)$	Use X’s AES key to encrypt M
$D_{SK_{X}}(M)$	Use X’s AES key to decrypt M
$Puk_{X}$	The public key of party X
$Prk_{X}$	The private key of party X
$E_{Puk_{X}}(M)$	Use X’s public key to encrypt M
$E_{Prk_{X}}(M)$	Use X’s private key to decrypt M
h(.)	The one-way hash function
τ	Valid timestamp interval
$M_{SUBMIT}$	The submitted registration information
$A\overset{?}{=}B$	Verify whether A is equal to B
